# Altitudinal biodiversity patterns of seed plants along Gongga Mountain in the southeastern Qinghai–Tibetan Plateau

**DOI:** 10.1002/ece3.5483

**Published:** 2019-08-07

**Authors:** Kuiling Zu, Ao Luo, Nawal Shrestha, Bo Liu, Zhiheng Wang, Xiangyun Zhu

**Affiliations:** ^1^ State Key Laboratory of Systematic and Evolutionary Botany, Institute of Botany Chinese Academy of Sciences Beijing China; ^2^ University of Chinese Academy of Sciences Beijing China; ^3^ Institute of Ecology, College of Urban and Environmental Sciences and Key Laboratory for Earth Surface Processes of the Ministry of Education Peking University Beijing China; ^4^ Minzu University of China Beijing China; ^5^ Southeast Asia Biodiversity Research Institute Chinese Academy of Sciences Nay Pyi Taw Myanmar

**Keywords:** elevational gradients, phylogenetic diversity, phylogenetic structure, species diversity

## Abstract

The mechanisms underlying elevation patterns in species and phylogenetic diversity remain a central issue in ecology and are vital for effective biodiversity conservation in the mountains. Gongga Mountain, located in the southeastern Qinghai–Tibetan Plateau, represents one of the longest elevational gradients (ca. 6,500 m, from ca. 1,000 to 7,556 m) in the world for studying species diversity patterns. However, the elevational gradient and conservation of plant species diversity and phylogenetic diversity in this mountain remain poorly studied. Here, we compiled the elevational distributions of 2,667 native seed plant species occurring in Gongga Mountain, and estimated the species diversity, phylogenetic diversity, species density, and phylogenetic relatedness across ten elevation belts and five vegetation zones. The results indicated that species diversity and phylogenetic diversity of all seed plants showed a hump‐shaped pattern, peaking at 1,800–2,200 m. Species diversity was significantly correlated with phylogenetic diversity and species density. The floras in temperate coniferous broad‐leaved mixed forests, subalpine coniferous forests, and alpine shrublands and meadows were significantly phylogenetically clustered, whereas the floras in evergreen broad‐leaved forests had phylogenetically random structure. Both climate and human pressure had strong correlation with species diversity, phylogenetic diversity, and phylogenetic structure of seed plants. Our results suggest that the evergreen broad‐leaved forests and coniferous broad‐leaved mixed forests at low to mid elevations deserve more conservation efforts. This study improves our understanding on the elevational gradients of species and phylogenetic diversity and their determinants and provides support for improvement of seed plant conservation in Gongga Mountain.

## 1 INTRODUCTION

Understanding elevational gradients in species and phylogenetic diversity not only provides important insights for the development of a general theory on species diversity patterns (Lomolino, 2001), but is also vital for effective biodiversity conservation in the mountains, and hence remains a central issue in ecology (Bhattarai & Vetaas, 2003; Cardinale et al., 2012). Different elevational patterns in species diversity have been observed: monotonic decrease with elevation, hump‐shaped patterns, and a plateau in species diversity followed by a species diversity decline (Rahbek, 2005). While many previous studies have explored the elevational gradients in species diversity, elevational changes in the other dimensions of biodiversity, such as phylogenetic diversity (PD), remain much less studied.

Phylogenetic diversity quantifies the combined phenotypic or genetic diversity across species, reflecting the evolutionary history of a set of taxa (Cadotte & Davies, 2016; Davies et al., 2007). A most commonly used measure of phylogenetic diversity is evaluated as the total branch lengths of the phylogeny linking species in an area (Faith, 1992). Phylogenetic diversity enhances our understanding of the influences of evolutionary history on biodiversity maintenance and provides a vital dimension of biodiversity for conservation (Tucker et al., 2017). Phylogenetic information has been used in ecology and biogeography at very large scales to detect the nonrandom distribution of lineages in relation to spatial and environmental gradients (Devictor et al., 2010) and to prioritize conservation of species assemblages that have a distinct evolutionary history relative to others in the region (Gonzálezcaro, Umaña, Álvarez, Stevenson, & Swenson, 2014). Both species and phylogenetic diversity have drawn strong attention from ecologists, and comparing the patterns of species and phylogenetic diversity is essential to understand the evolutionary mechanisms of species diversity patterns. In addition, the relationship between biodiversity and ecosystem functions has become one of the central topics in ecological research (Cardinale et al., 2012; Loreau et al., 2001). Recent studies suggest that phylogenetic diversity is a better index explaining the biodiversity–ecosystem function relationships (Kembel & Hubbell, 2006; Zhang, Chen, & Reich, 2012). Therefore, combining the patterns of species and phylogenetic diversity is also vital to understand the relationship between biodiversity and ecosystem functions.

Understanding the drivers of species diversity changes along elevational gradients remains one of the major challenges of biogeographical research (Acharya, Chettri, & Vijayan, 2011; Kessler, 2009). Many hypotheses have been proposed to explain the elevational gradients in species diversity in terms of the effects of area (Rahbek, 1995; Wang, Tang, & Fang, 2007), climate (Hawkins, Diniz‐Filho, Jaramillo, & Soeller, 2007; Li, Zhu, Niu, & Sun, 2014), and macroevolution (e.g., niche conservatism; Hawkins et al., 2007; Kozak & Wiens, 2010). A growing body of evidence has confirmed that area may influence species diversity changes with elevation (Bachman, Baker, Brummitt, Dransfield, & Moat, 2004; Fu et al., 2007; McCain, 2009; Wang et al., 2007; Wu et al., 2013). Previous studies have demonstrated that contemporary climate including mean annual temperature (MAT), annual precipitation (AP), and their combination (annual evapotranspiration, AE) has considerable explanatory power for elevational patterns of species diversity (Hawkins et al., 2007; Hawkins, Diniz‐Filho, & Soeller, 2005; McCain, 2009). For instance, species diversity and phylogenetic structure of vascular plants in the Qinghai–Tibetan Plateau had strong associations with temperature and precipitation (Yan, Yang, & Tang, 2013). Similarly, a strong quadratic relationship between phylogenetic structure and climate was observed in the Hengduan Mountains (Li et al., 2014). Compared with the elevational patterns in species diversity, the effects of these factors on the elevational patterns in phylogenetic diversity have been much less studied.

Fossil and molecular evidence has shown that many mountains may have acted as cradle for the evolution of biodiversity (Hughes & Atchison, 2015) and/or as refugia during climate change (Perrigo, Hoorn, & Antonelli, 2019; Tang et al., 2018) and hence generally contain higher biodiversity than surrounding lowlands. Therefore, in many regions, mountains have been identified as prioritized areas for biodiversity conservation (Tang, Wang, Zheng, & Fang, 2006). For example, several mountains, such as the Andes, the Hengduan Mountains, and the Caucasus, have been identified as biodiversity hot spots for conservation (Favre et al., 2015) and attracted many conservation resources. However, the threats to biodiversity in mountains induced by human activities (such as tourism and forestry industry) are escalating, especially in the developing countries (Bookbinder, Dinerstein, & Rijal, 1998; Li, Kraft, Yu, & Li, 2015; Tang et al., 2006; Wilson, 1992). For example, recent studies suggest that more than 33.2 million km^2^ or 85.7% of mountainous areas globally (outside Antarctica) are under influences induced by human activities (David & Bomhard, 2012). These findings suggest that biodiversity in mountains is facing increasingly heavy anthropogenic disturbances and threats (Nogués‐Bravo, Araújo, Romdal, & Rahbek, 2008; Wang, Fang, Tang, & Lin, 2011; White & Kerr, 2007). Therefore, it is essential to evaluate the effects of human disturbance on the elevational patterns in species and phylogenetic diversity for effective biodiversity conservation and sustain the ecosystem functioning in economically impoverished but biologically rich mountains. In addition, while many studies have focused on the conservation of species diversity, the evolutionary history that maintains and generates biodiversity has also been recognized as an important dimension of biodiversity that needs to be considered in conservation planning (Winter, Devictor, & Schweiger, 2013). However, quantitative analyses are needed to reveal the evolutionary diversity of seed plants in poorly known but important mountains.

Gongga Mountain (Mt. Gongga) with a broad elevational gradient (from ca. 1,000 to 7,556 m a.s.l.), located between the southeastern edge of the Qinghai–Tibetan Plateau (QTP) and the Sichuan Basin, is one of the highest mountains in the Himalaya–Hengduan Mountains region and southwest China. This mountain is located in the area that has been widely recognized as one of the global biodiversity hot spots (Myers, Mittermeier, Mittermeier, Fonseca, & Kent, 2000; Wu et al., 2013). Previous studies suggest that Mt. Gongga is the cradle and refuge of plant diversity in southwest China, as reflected by the fact that it contains high plant diversity and many relict plant species (Liu & Yin, 1985). Mt. Gongga can be regarded as a “natural laboratory” for studying the drivers of elevational gradients in species and phylogenetic diversity. Plant distributions in mountainous areas are more easily affected by climate change. For instance, montane species may shift upwards and their distributional range may be lost under climate warming (He et al., 2019; Liang et al., 2018). Exploring the elevational patterns in plant species and phylogenetic diversity in Mt. Gongga and their drivers would contribute to the conservation of plant diversity in this region. Here, we aimed to (a) characterize the elevational patterns in species diversity of all, endemic, endangered, and economically valuable seed plant species; (b) estimate elevational patterns in phylogenetic diversity and phylogenetic structure; (c) explore the drivers of these patterns. These findings will give a better understanding of phylogenetic diversity patterns along elevation and further improve our insight on the drivers of the elevational patterns in species and phylogenetic diversity. This work will also provide more support for biodiversity conservation in Mt. Gongga.

## 2 MATERIAL AND METHODS

### 2.1 Study area

**Figure 1:**
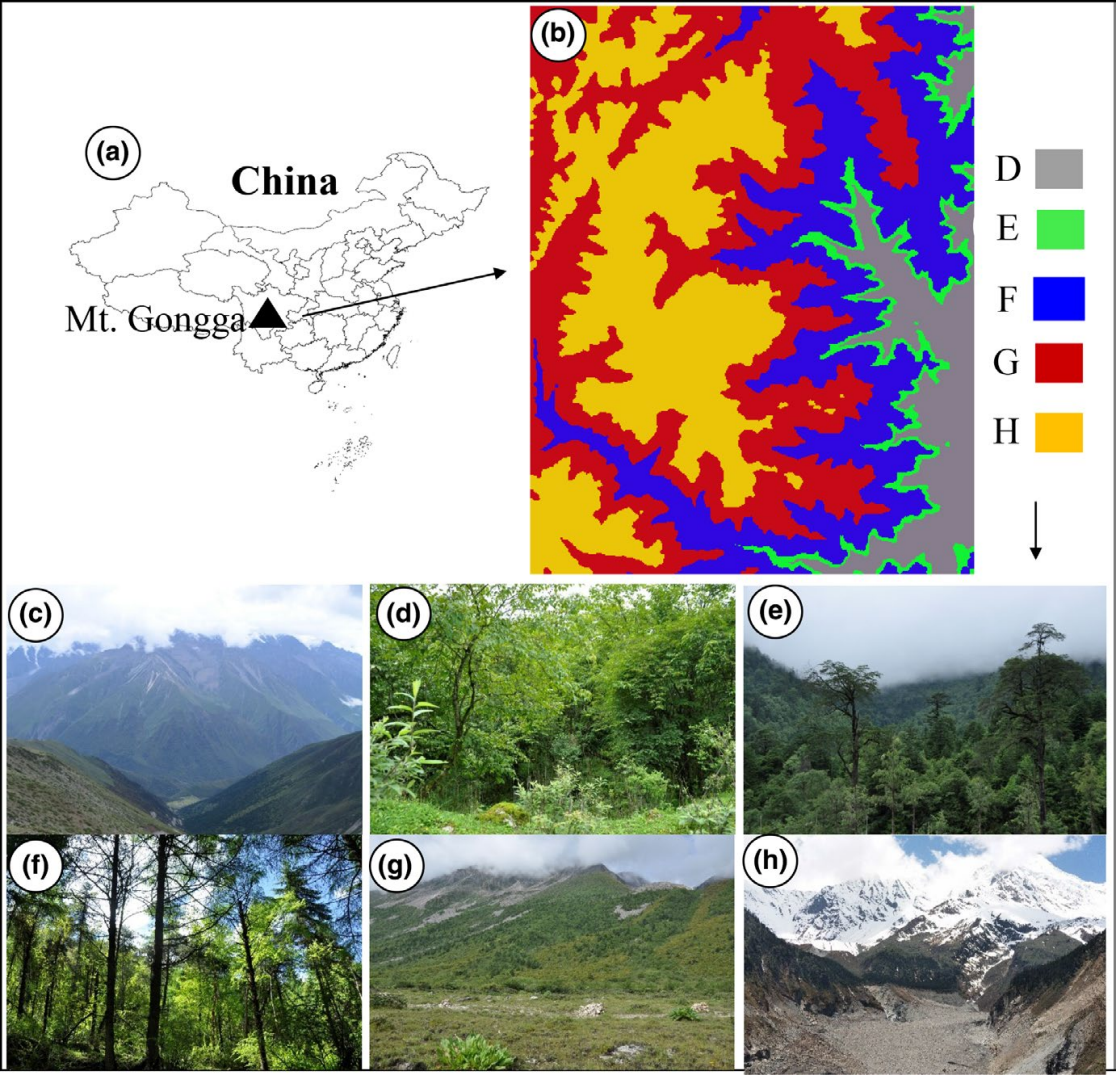
Location and vegetation zones of Mt. Gongga in the southeastern Qinghai–Tibetan Plateau. The location (a), altitude (b), and distant overview (c) of Mt. Gongga; (d) evergreen broad‐leaved forest (EBF); (e) temperate coniferous broad‐leaved mixed forest (CBM); (f) subalpine coniferous forest (SC); (g) alpine shrub and meadow (ASM); (h) alpine scree and nival zone (ASN). Photographs were taken by Kuiling Zu (d, e, f, g, h) and Zhi Yang (University of Chinese Academy of Sciences) (c)

Gongga Mountain is located in the western part of the Sichuan province in China between 29°21′N–29°54′N and 101°44′–102°10′E (Figure 1). It is the transitional zone between the eastern edge of the Qinghai–Tibet Plateau and the Sichuan basin and is also the most important mountain of the Hengduan Mountains range that forms the core of the south‐central China biodiversity hot spot (Myers et al., 2000). It is located close to the boundary of “Tanaka‐Kaiyong Line” and is therefore considered a key biodiversity area. It lies along the divide between the flora of “the China‐Himalayan region” and “the China–Japan region” (Shen, Liu, & Wu, 2004). The elevation in Mt. Gongga ranges from 1,000 to 7,556 m across the region from north to south. The annual precipitation is about 1,952 mm with a relative humidity of up to 76%; the temperature ranges between −14.0°C at the alpine region and 23.2°C at the foothill. Both the climate zones and vegetation zones change accordingly with elevation (Liu & Yin, 1985), leading to a rich diversity of plant community types, including evergreen broad‐leaved forest (EBF), temperate coniferous broad‐leaved mixed forest (CBM), subalpine coniferous forest (SC), alpine shrub and meadow (ASM), alpine scree, and nival zone (ASN; Table 1, Figure 1).

**Table 1:** Elevation ranges, representative species, elevation belts, and area surfaces for the five vegetation zones of Mt. Gongga

Community type	Elevation (m)	Dominant species	Elevation belts	Area (km^2^)
Evergreen broad‐leaved forest	1,000–2,200	*Cyclobalanopsis glauca*, *C. oxyodon*	H1, H2, H3	527.70
Temperate coniferous broad‐leaved mixed forest	2,200–2,500	*Tsuga chinensis*, *Acer flabellatum*	H4	230.32
Subalpine coniferous forest	2,500–3,600	*Picea brachytyla*, *Abies fabri*	H5, H6, H7	763.06
Alpine shrub and meadow	3,600–4,600	*Rhododendron cephalantum*, *Kobresia pygmaea*	H7, H8, H9	1,031.71
Alpine scree and nival zone	above 4,600	*Saussurea quercifolia*	H10	645.23

Note

The five vegetation zones of Mt. Gongga are based on Liu and Yin (1985).

### 2.2 Data sources of species distributions

We created a comprehensive checklist of seed plants, which contains 2,667 native species belonging to 677 genera and 137 families based on field investigation and specimen collection until 2018 (Table 2). Elevational data were compiled from a total of 8,115 specimen records (8.6% from field specimen collection in 2018 and 91.4% from other years). Specimen records were obtained from the *Biodiversity of the Hengduan Mountains and adjacent areas of south‐central China database* (http://hengduan.uh.harvard.edu/fieldnotes), *National Specimen Information Infrastructure* (http://www.nsii.org.cn/2017/home.php), and the *Chinese Virtual Herbarium* (http://www.cvh.org.cn/cms/). We standardized species names using the Taxonomic Name Resolution Service (tnrs.iplantcollaborative.org/TNRSapp.html) and Asian Plant Synonym Lookup (phylodiversity.net/fslik/synonym_lookup.htm).

**Table 2:** Numbers of economically valuable, endangered and endemic plants in Mt. Gongga

Category	Species	Genus	Family
Total	2,667	677	137
Economic plants^a^	150	112	59
IUCN‐CR^b^	11	8	8
IUCN‐EN^c^	26	17	11
IUCN‐VU^d^	64	50	33
Endemic plants^e^	67	43	21

a: Liu and Yin (1985); b‐d: Qin et al. (2017); e: Huang et al. (2014).

The patterns in species diversity of endemic (Huang, Ma, & Chen, 2014), endangered, and economically valuable plants (Liu & Yin, 1985) were estimated to explore the elevational patterns in seed plant resources in this region. The list of threatened plants in Mt. Gongga was compiled from *China Biodiversity Red List* (China's State Forestry Administration & the Institute of Botany, Chinese Academy of Sciences, 2013). Threatened plants were referred to as critically endangered (CR), endangered (EN), and vulnerable (VU) species according to the IUCN red list categories and were obtained from Qin et al. (2017).

### 2.3 Data sources of climate variables and human footprint

The climate data were extracted from the *WorldClim* database (http://www.worldclim.org) and *CGIAR Consortium for Spatial Information* (http://www.cgiar-csi.org/) at the spatial resolution of 30‐arc‐second (c. 1 skm^2^; Hijmans, Cameron, Parra, Jones, & Jarvis, 2005; Trabucco & Zomer, 2019).The extracted climate data included annual mean temperature (AMT), annual precipitation (AP), mean diurnal range of temperature (MDR), annual evapotranspiration (AE), and temperature annual range (TAR). Human footprint (HF) data during the period of 1995–2004 were obtained from Last of the Wild (v2), ranging from 0 to 100 (higher number indicate higher human influence; LWP‐2, 2005). The area of each elevational belt was calculated in ArcGIS 10.2 (ESRI) using the digital elevation model (Wang, Chen, & Fang, 2004; Figure S1). The relationship between phylogenetic indices and areas as well as the relationships between phylogenetic indices and climate factors were examined using the simple ordinary least squares (OLS) for multiple regressions. For the polynomial regression, the Bayesian Information Criterion (BIC) and *R* ^2^ were calculated, and were used to make a comparison among the first‐order, second‐order term, and third‐order polynomial models (Li et al., 2014). The results with significant statistical relationships are shown in Table S1.

### 2.4 Phylogenetic reconstruction

A phylogenetic tree for all the seed plants in Mt. Gongga was reconstructed using the online bioinformatics tool Phylomatic (Webb & Donoghue, 2005), and the phylogenetic tree from Zanne et al. (2014), which contained 32,223 flowering plant species. This phylogeny was constructed by seven DNA regions (18S rDNA, 26S rDNA, ITS, matK, rbcL, atpB, and trnL‐F), and its topology was constrained mainly by the phylogeny reconstructed by Soltis et al. (2011) and APG III. The divergence time was calibrated with 39 commonly used fossils. This phylogeny represents the current understanding about the relationships between major clades of flowering plants and has been widely used in previous studies (e.g., Diaz, Harmon, Sugawara, Miller, & Pennell, 2019).

### 2.5 Species diversity, phylogenetic diversity, and phylogenetic structure along elevation

We divided the study region into 10 elevational belts at 400 m vertical intervals, H1: 1,000–1,400 m, H2: 1,400–1,800 m, H3: 1,800–2,200 m, H4: 2,200–2,600 m, H5: 2,600–3,000 m, H6: 3,000–3,400 m, H7: 3,400–3,800 m, H8: 3,800–4,200 m, H9: 4,200–4,600 m and H10: 4,600–5,000 m (Table 1). Preliminary analyses based on elevational intervals of 100, 200, and 600 m demonstrated similar patterns in species diversity as those based on the 400‐m belts. The area below 1,000 m a.s.l. is highly disturbed by farming and deforestation, which was not well investigated according to the field inventory literature. The area above 5,000 m a.s.l. has few species and was not well investigated. Therefore, we restricted our analyses from elevations 1,000 to 5,000 m a.s.l.

Based on the interpolated ranges of species, we calculated the species diversity for each elevational belt using the observed species distribution in our field surveys. Moreover, species density (*D*) of each belt was calculated following Vetaas and Grytnes (2002) and Wang et al. (2007) as *D* = *S*/ln (*A*), where *S* is the number of species and *A* is the area of each belt.

Faith's phylogenetic diversity (Faith, 1992) was used to estimate the phylogenetic diversity of seed plants within each elevational belt. As species diversity is generally strongly positively related to PD, PD measurements should be standardize to the observed species diversity to evaluate the contribution of evolutionary history to phylogenetic diversity after species diversity was accounted for. We calculated the standard effect size of phylogenetic diversity (PD_SES_) by dividing the difference between the observed and expected PD by the standard deviation of the null distribution (Webb, Ackerly, & Kembel, 2008) as follows:

$${\text{PD}}_{\text{SES}}=\left({\text{PD}}_{\text{observed}}-{\text{PD}}_{\text{randsample}}\right)/\text{sd}\left({\text{PD}}_{\text{randsample}}\right)$$

PD_observed_ is the observed value and PD_randsample_ is the mean value of 999 random samplings with equivalent species diversity. sd is the standard deviation of a random sample. PD_SES_ > 0 means that PD_observed_ is higher than the random expectation, while PD_SES_ < 0 means that PD_observed_ is lower than the random expectation. The PD_SES_ value of each elevation belts was calculated in R 3.3.3 software using the “picante” package. The sample file was first converted to matrix, and PD_SES_ was calculated using the generated phylo file and the null model by repeating 999 times.

The phylogenetic structure of each belt was calculated using two indices: the net relatedness index (NRI) based on the mean phylogenetic distance (MPD); and the nearest species index (NTI) based on the mean nearest taxon distance (MNTD). Among them, MPD indicates the average phylogenetic relatedness between all possible pairs of species in one belt, and MNTD indicates the mean phylogenetic relatedness between each species and its nearest relative in one belt (Webb, Ackerly, McPeek, & Donoghue, 2002). Based on MPD and MNTD, we calculated NRI and NTI indices as follows:

$$\text{NRI}=-1\times \left({\text{MPD}}_{\text{observed}}-{\text{MPD}}_{\text{randomized}}\right)/{\text{sdMPD}}_{\text{randomized}}$$

MPD_randomized_ and sdMPD_randomized_, respectively, represented the mean and standard deviation of MPD estimated using 999 randomly sampled communities with given species diversity. Similarly, MNTD_randomized_ and sdMNTD_randomized_, respectively, represented the mean and standard deviation of MNPD estimated using 999 randomly sampled communities with given species diversity. NRI and NTI values >1.96 reflect significant phylogenetic clustering, while NRI and NTI values <−1.96 reflect significant phylogenetic overdispersion (Webb et al., 2002). NRI and NTI values between −1.96 and 1.96 suggest that phylogenetic structures are not significantly different from random expectation.

All these analyses were performed in Phylocom 4.2 (Webb et al., 2008) and R 3.3.3 software using the “picante” package (Kembel et al., 2010).

## 3 RESULTS

### 3.1 Elevational gradients in species diversity, species density, and phylogenetic diversity

**Figure 2:**
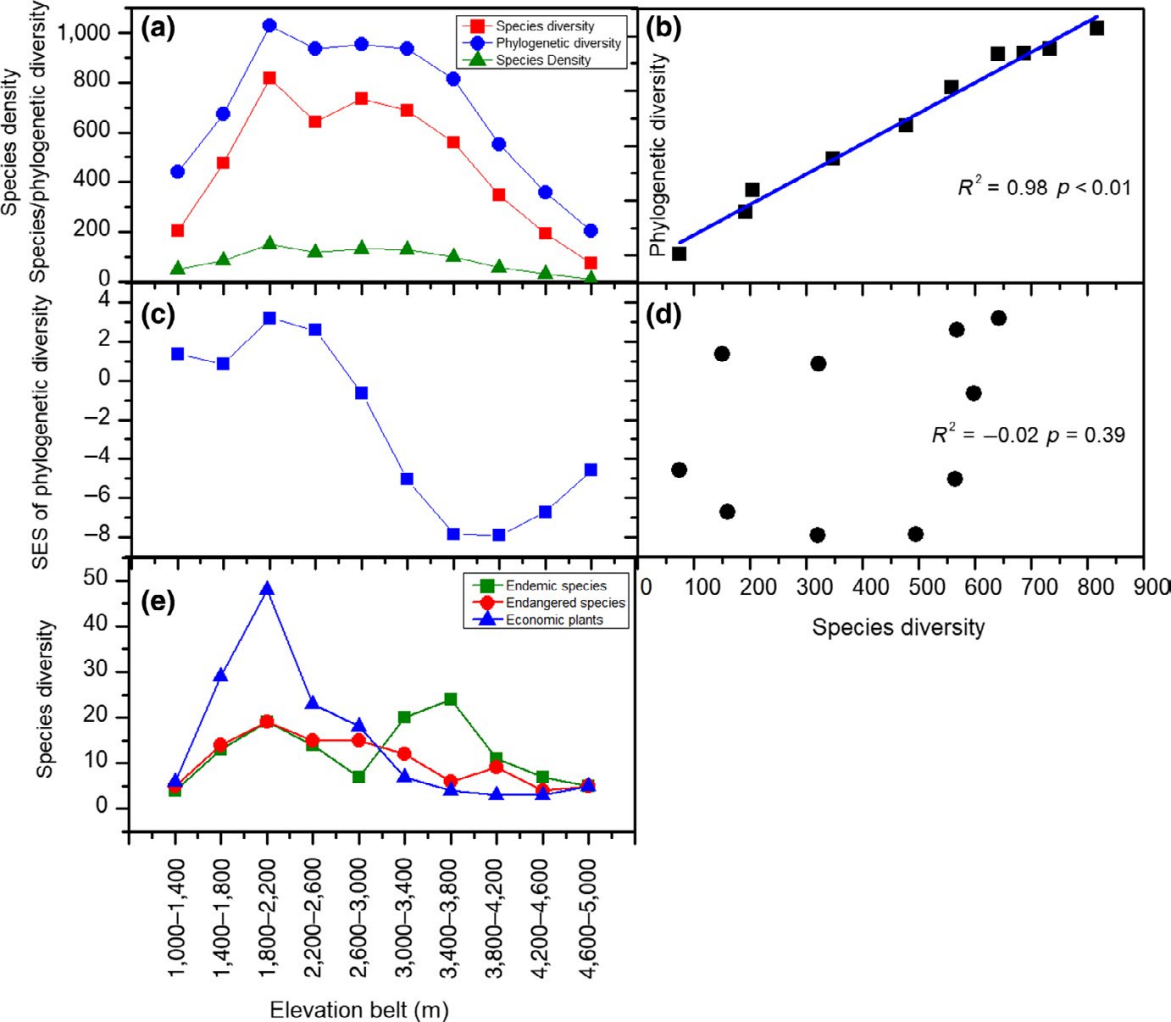
The elevational patterns in species diversity, species density, and phylogenetic diversity of seed plants in Mt. Gongga and the relationships between phylogenetic and species diversity. (a) The patterns of species diversity, species density, and phylogenetic diversity along the elevational gradient. (b) The relationship between species diversity and phylogenetic diversity. (c) The pattern of standardized effect size of PD (PD_SES_) along the elevational gradient. (d) The relationship between species diversity and PD_SES_. (e) Species diversity of endangered, endemic, and economically valuable plants

Species diversity ranged from 74 to 818 species per elevational belt, with a mean value of 474 species. The species density ranged from 12.5 to 151.0 per elevation belt, and the mean value was 87.4 (Figure 2a). Species and phylogenetic diversity (PD_observed_) had similar elevational patterns (*R* ^2^ = .98, *p* < .01; Figure 2b): both showed hump‐shaped patterns along elevational gradient with peaks occurring between 1,800 and 2,200 m a.s.l., which is the upper boundary of evergreen broad‐leaved forest.

PD_SES_ showed a hump‐shaped pattern along elevational gradient. PD_observed_ was higher than the random expectation given the level of species diversity at low elevations (1,000–2,600 m), but lower than the random expectation at high elevations (above 2,600 m). We found that the PD_SES_ was not correlated with species diversity (*R* ^2^ = −.02, *p* = .39; Figure 2c,d).

### 3.2 Elevational gradients in diversity of endangered, endemic, and economically valuable species

A large number of endangered plants, endemic plants, and economically valuable species exist in Mt. Gongga. In total, 150 economically valuable species, 67 endemic species and 101 endangered species of plants were recorded (Table 2). The endemic species diversity was the highest at the elevations of 3,000–3,800 and 1,800–2,200 m. The species diversity of both the endangered species and economically valuable species was the highest at the elevations of 1,800–2,200 m (Figure 2e).

### 3.3 Elevational gradients in phylogenetic structures of seed plants

**Figure 3:**
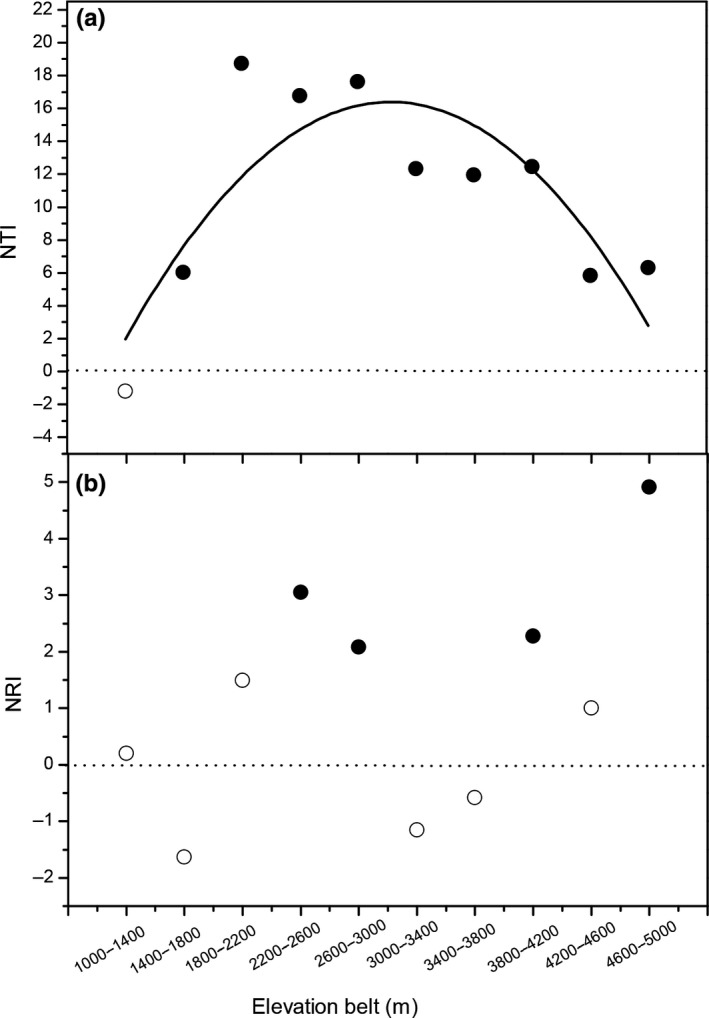
The elevational patterns in nearest species index (NTI) (a) and the net relatedness index (NRI) (b) of seed plants in Mt. Gongga. Empty circles represent phylogenetically random structure and solid black dots represent phylogenetic clustering

Similar to the elevational gradient in species diversity, NTI also showed a hump‐shaped pattern along the elevational gradient with a peak at 1,800–2,200 m. The NTI values were >1.96 in the elevational belts above 1,800 m, indicating phylogenetic clustering, but were between −1.96 and 0 in the elevational belt of 1,000–1,400 m, indicating random phylogenetic structure. In contrast, NRI increased with the elevation and was >1.96 in the elevational belts of 2,200–3,000, 3,800–4,200, and 4,600–5,000 m, indicating phylogenetic clustering. However, NRI was between −1.96 and 1.96 in the elevational belts of 1,400–1,800, 3,000–3,800, and 4,200–4,600 m, indicating random phylogenetic structure. Several community types, for example, temperate coniferous broad‐leaved mixed forest, subalpine coniferous forest, and the alpine shrublands and meadows were significantly phylogenetically clustered, while evergreen broad‐leaved forest had phylogenetically random structure (Figure 3).

### 3.4 Determinants of species and phylogenetic diversity and phylogenetic structures of seed plants along elevational gradient

**Figure 4:**
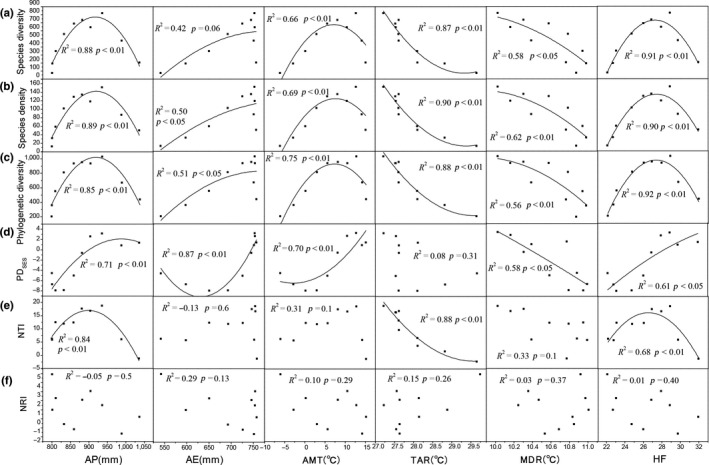
The changes in species diversity (a), species density (b), phylogenetic diversity (c), PD_SES_ (d), NTI (e), and NRI (f) as functions of different factors of climate and human activities. To account for the nonlinear relationships, quadratic polynomial models were used. AE, annual evapotranspiration; AMT, annual mean temperature; AP, annual precipitation; HF, human footprint; MDR, mean diurnal range of temperature; TAR, temperature annual range

Species diversity, species density and phylogenetic diversity had similar relationships with the environmental factors. AP, AMT, and human influence index significantly explained high proportions of variations in species diversity, species density, and phylogenetic diversity. With the increase in AP, species diversity (*R* ^2^ = .88, *p* < .01), species density (*R* ^2^ = .89, *p* < .01), and phylogenetic diversity (*R* ^2^ = .85, *p* < .01) first increased, reached a peak at AP = 920 mm and then decreased. Similarly, with the increase in AMT, species diversity (*R* ^2^ = .66, *p* < .01), species density (*R* ^2^ = .69, *p* < .01), and phylogenetic diversity (*R* ^2^ = .75, *p* < .01) first increased to a peak value at AMT = 8°C and then decreased. With the increase in human influence index, species diversity (*R* ^2^ = .91, *p* < .01), species density (*R* ^2^ = .90, *p* < .01), and phylogenetic diversity (*R* ^2^ = .92, *p* < .01) first increased, reached a peak at human influence index = 27 and then decreased. Both MDR and TAR had negative effects, while AE had positive effects on species diversity, species density, and phylogenetic diversity (except that species diversity had no relationship with AE; Figure 4a,b,c). PD_SES_ had positive correlations with AP, AMT, and HF, but a negative correlation with MDR. PD_SES_ decreased first and then increased with the increase in AE (*R* ^2^ = .87, *p* < .01), and PD_SES_ had no correlation with AMT (Figure 4d).

Among all the environmental factors, AP (*R* ^2^ = .84, *p* < .01) and human influence index (*R* ^2^ = .68, *p* < .01) had the highest explanatory powers for NTI (Figure 4d), while none of them were significantly correlated with NRI (Figure 4f). With the increase in area, the species diversity, phylogenetic diversity, and NTI all increased first and then decreased (Figure S2).

## 4 DISCUSSION

### 4.1 Patterns and determinants of seed plant diversity along Mt. Gongga

The hump‐shaped pattern of species diversity recovered in the current study is consistent with the findings of previous studies on the species diversity of seed plants along elevational gradients in other regions (e.g., Gaoligong Mountains and Dulong Valley in southeast Qinghai–Tibetan Plateau, Li et al., 2015; Wang et al., 2007). There was a strong positive correlation between species and phylogenetic diversity along the elevational gradient in Mt. Gongga, which is consistent with previous findings. For instance, Rodrigues and Gaston (2002) found a high spatial congruence between phylogenetic and generic diversity of birds in northwest South Africa. Similarly, Zhou et al. (2018) found that the species diversity, species density, and phylogenetic diversity were positively correlated with each other across different vegetation zones in Mount Kenya.

The result that both species and phylogenetic diversity correlated well with environmental factors along the elevational gradients is consistent with previous findings on breeding bird species in the Hengduan Mountains (Wu et al., 2013). In particular, our analysis showed that there is a strong correlation between AP and diversity, which suggest that precipitation is the predominant environmental factor on Mt. Gongga. Similar results have been observed in alpine grasslands that precipitation is the predominant environmental factor on the Qinghai–Tibetan Plateau (Wu, Shen, & Zhang, 2014). Such a higher explanatory power of precipitation supports the water–energy hypothesis (O'Brien, 1998). The hypotheses proposed to the dominant effect of available water on species diversity patterns (Wang et al., 2011). Our results together with previous findings suggest that water availability may be the limiting factor for species diversity patterns in Mt. Gongga.

With the increased inhuman activities, the species diversity and phylogenetic diversity first increased and then decreased, supporting the view that a unimodal model explains the relationship between vascular plant species diversity and human disturbance. This pattern is consistent with the intermediate disturbance hypothesis (Bongers, Poorter, Hawthorne, & Sheil, 2009; Mayor, Cahill, He, Sólymos, & Boutin, 2012). This result may suggest that disturbance may significantly contribute to the hump‐shaped elevational patterns in species and phylogenetic diversity in the subtropical mountains. Previous studies have shown that lower regions in the mountainous areas at the global scale are easily affected by the settlements and exploitation of forest resources is very high in these areas (Nogués‐Bravo et al., 2008). The species of economic interests growing in the forest areas close to villages in Mt. Gongga are subjected to overexploitation (Xu & Wilkes, 2004), and hence are seriously threatened. Strong human disturbances at low elevation could be one of the reasons for the low species and phylogenetic diversity in this region. Our findings suggest that both climate and human pressure have important effects on the species and phylogenetic diversity along the elevational gradients. Because the diversity patterns are driven by multiple explanatory factors, finer studies at local scales would further improve our understanding of the determinants in this hot spot region.

### 4.2 Drivers of phylogenetic structures of seed plants

Both the values of NTI and NRI revealed two different kinds of phylogenetic structures (clustered and random) along the elevational gradient. The floras in temperate coniferous broad‐leaved mixed forests, subalpine coniferous forests, and the alpine shrublands and meadows were significantly phylogenetically clustered, whereas the floras in the evergreen broad‐leaved forests had phylogenetically random structure. Phylogenetic clustering occurred at cool high‐elevation environments, likely due to environmental filtering at high elevations. Closely related plant species have similar traits to survive in the harsh climate prevailing at high elevations, which could be one of the possible reasons for phylogenetic clustering in these regions (Li et al., 2014; Webb et al., 2002). Similarly, Graham, Parra, Rahbek, and Mcguire (2009) also found that phylogenetically clustered communities of humming birds in the Andes occurred in cool high‐elevation regions tropical. Our results and previous findings are in line with the phylogenetic niche conservatism hypothesis, which suggests that species in cold regions tend to be more phylogenetically related than those in warmer regions (Qian, Zhang, Zhang, & Wang, 2013).

The biogeographical events may also contribute to the modern spatial patterns of biodiversity (Svenning, Eiserhardt, Normand, Ordonez, & Sandel, 2015). For example, the repeated rise and fall of many glaciers along the altitude during the Quaternary may have shaped the contemporary patterns of species along the elevational gradient (Zhong, Qin, & Xu, 1979). The minimum snow line of Mt. Gongga was about 1,400 m during the Quaternary glaciations (2.56 Ma; Zhong et al., 1979). Our phylogenetic results show that seed plants distributed in the elevational belts of 1,400–1,800 and 3,000–3,800 m have negative NRI, which might be due to the colonization of species from other adjacent regions (Figure 3). Similar biogeographical events have been attributed to the richness pattern in the Dulong Valley Region and the Hengduan Mountains region (Li et al., 2015, 2014). It would be interesting to study further the divergence times and evolution history of the alpine plants in these regions.

### 4.3 Phylogenetic diversity in conservation planning

Understanding the evolutionary history of regional floras is useful to reveal the causes of contemporary biodiversity patterns, which lays the foundation for decision‐making in biodiversity conservation (Lu et al., 2018). Previous studies have shown that maximizing the conservation of phylogenetic diversity will in turn maximize the options within the flora for future diversification (Li et al., 2015). Biodiversity conservation should consider evolutionary processes that shape gradients of species diversity and the features of landscapes, which is recommended by more and more researchers (Carnicer, Brotons, Stefanescu, & Peñuelas, 2012; Forest et al., 2007; Rosauer, Laffan, Crisp, Donnellan, & Cool, 2009). Along this line, we suggest that evergreen broad‐leaved forest at low to mid elevations should be given more conservation attention because of the high phylogenetic diversity and high number of endemic and economically valuable plants in these communities.

Specifically, the maximum number of economically valuable and endangered species was found in the evergreen broad‐leaved forest at low to mid elevations. The endangered species are usually rare species and require wide conservation attention because they are more prone to extinction (Arponen, 2012). The protection of endangered rare species is often correlated with phylogenetic distinctiveness leading to the conservation of distinct species (Redding & Arne, 2007; Winter et al., 2013). Therefore, protection should be strengthened in the regions that have a large number of endangered rare plants. To our knowledge, biodiversity conservation at low to mid elevations (2,200–2,600 m) in Mt. Gongga has not received proper attention, and hence should be enhanced in the future. Therefore, reducing the overexploitation of economic species and protecting the endemic species should be urgent tasks in low to mid elevations in Mt. Gongga.

The data used in this study are only sufficient to address the conservation concerns on plant diversity in Mt. Gongga. Detailed studies on all organisms, including ferns, birds, and insects, will enable us to better understand the origin and evolution of the biodiversity in Mt. Gongga and the impact of global changes on alpine biodiversity, which will effectively improve biodiversity conservation in this region.

## CONFLICT OF INTEREST

No conflict of interest exits in the submission of this manuscript.

## AUTHOR CONTRIBUTIONS

KZ conceived and wrote the manuscript. AL extracted the data of environmental variables. NS and BL checked the data and gave scientific suggestions. ZW and XZ designed the research project and critically revised the text. All authors reviewed the manuscript.

## DATA AVAILABILITY STATEMENT

The data will be made available in the Dryad Digital Repository upon acceptance of the manuscript (https://doi.org/10.5061/dryad.739sh81). The authors are solely responsible for these data. Queries (other than absence of the material) should be directed to the corresponding author.
