# Supplementary material for: Altitudinal biodiversity patterns of seed plants along Gongga Mountain in the southeastern Qinghai–Tibetan Plateau
Source: Ecol Evol. 2019 Aug 7;9(17):9586–96. doi: 10.1002/ece3.5483 (PMC6745871; doi:10.1002/ece3.5483)
Supplement: Supplementary file 1 [file ECE3-9-9586-s001.docx]

**Supplementary material for:**

*Altitudinal biodiversity patterns of seed plants along Gongga Mountain in the southeastern Qinghai-Tibetan Plateau*

**Table S1** Regression statistics for species diversity and phylogenetic relatedness related to underlying factors. Black fonts show the most appropriate model.

| Category | Response  variable | Polynomial order | R² | p-value | BIC |
| --- | --- | --- | --- | --- | --- |
| Species diversity | Annual Mean Temperature | 1 | 0.18278 | 0.12081 | 111.34082 |
|  |  | 2 | 0.65638 | 0.00987 | 101.34184 |
|  |  | **3** | **0.88798** | **1.17E-04** | **86.76873** |
|  | Annual Precipitation | 1 | -0.08272 | 0.59153 | 114.15413 |
|  |  | 2 | 0.87819 | 2.62E-04 | 90.971416 |
|  |  | **3** | **0.89444** | **7.42E-04** | **87.99804** |
|  | Mean Diurnal Range | **1** | **0.61809** | **0.00426** | **103.73369** |
|  |  | 2 | 0.5773 | 0.02038 | 103.41317 |
|  |  | 3 | 0.47993 | 0.00503 | 102.12131 |
|  | Annual Evapotranspiration | **1** | **0.46048** | **0.01853** | **107.1886** |
|  |  | 2 | 0.41884 | 0.06209 | 106.5968 |
|  |  | 3 | 0.44553 | 0.09382 | 104.58513 |
|  | Temperature Annual Range | 1 | 0.75814 | 0.0006424 | 99.16555 |
|  |  | **2** | **0.86849** | **3.42E-04** | **91.7371** |
|  |  | 3 | 0.84987 | 0.00211 | 91.520076 |
|  | Area | 1 | 0.00514 | 0.33625 | 113.30777 |
|  |  | 2 | 0.74155 | 9.18E-04 | 95.128754 |
|  |  | **3** | **0.78012** | **0.0065** | **95.33576** |
|  | Human footprint | **1** | -0.0479 | 0.465 | 109.8829 |
|  |  | **2** | **0.88096** | **2.41529E-4** | **86.79707048** |
|  |  | 3 | 0.87037 | 0.00137 | 86.10751 |
| Phylogenetic diversity | Annual Mean Temperature | 1 | 514325.96 | 0.09418 | -33.36215 |
|  |  | 2 | 0.75155 | 0.00317 | 100.3666 |
|  |  | **3** | **0.90144** | **6.05E-04** | **89.57916** |
|  | Annual Precipitation | 1 | -0.06731 | 0.52926 | 116.27852 |
|  |  | 2 | 0.85463 | 4.86E-04 | 95.00733 |
|  |  | **3** | **0.91126** | **4.43E-04** | **88.53017** |
|  | Mean Diurnal Range | **1** | **0.59048** | **0.00572** | **106.6995** |
|  |  | 2 | 0.55708 | 0.024 | 106.14825 |
|  |  | 3 | 0.48912 | 0.0745 | 106.03411 |
|  | Annual Evapotranspiration | **1** | **0.53113** | **0.01014** | **108.0528** |
|  |  | 2 | 0.51212 | 0.03366 | 107.11493 |
|  |  | 3 | 0.55035 | 0.04184 | 104.75747 |
|  | Temperature Annual Range | 1 | 0.81356 | 2.22E-04 | 98.830738 |
|  |  | **2** | **0.88212** | **2.33E-04** | **92.91088** |
|  |  | 3 | 0.8625 | 0.00163 | 92.909233 |
|  | Area | 1 | 0.05591 | 0.25076 | 115.05174 |
|  |  | 2 | 0.72664 | 0.00443 | 101.32232 |
|  |  | **3** | **0.77748** | **0.00673** | **97.72271** |
|  | Human footprint | 1 | 0.04957 | 0.26002 | 115.1187 |
|  |  | **2** | **0.91895** | **6.29E-05** | **89.16509** |
|  |  | 3 | 0.91659 | 3.68E-04 | 87.91069 |
| NRI | Annual Mean Temperature | 1 | 0.12318 | 0.1708 | 15.202911 |
|  |  | 2 | 0.09778 | 0.28946 | 14.153124 |
|  |  | 3 | 0.34337 | 0.1501 | 9.4342577 |
|  | Annual Precipitation | 1 | 0.0703 | 0.231 | 15.788463 |
|  |  | 2 | -0.05876 | 0.50675 | 15.753097 |
|  |  | 3 | -0.18779 | 0.68061 | 15.36151 |
|  | Mean Diurnal Range | 1 | -0.1236 | 0.92285 | 17.682745 |
|  |  | 2 | 0.03467 | 0.36674 | 14.829215 |
|  |  | 3 | -0.10187 | 0.57423 | 14.610632 |
|  | Annual Evapotranspiration | 1 | 0.17375 | 0.12741 | 14.608864 |
|  |  | 2 | 0.28728 | 0.12683 | 11.795454 |
|  |  | 3 | 0.17362 | 0.2791 | 11.733565 |
|  | Temperature Annual Range | 1 | 0.25809 | 0.14595 | 12.196752 |
|  |  | 2 | 0.25809 | 0.14595 | 12.196752 |
|  |  | 3 | 0.13541 | 0.31418 | 12.185541 |
|  | Area | 1 | 0.17375 | 0.12741 | 14.608864 |
|  |  | 2 | -0.00927 | 0.42856 | 15.274319 |
|  |  | 3 | -0.01172 | 0.46795 | 13.757059 |
|  | Human footprint | 1 | 0.08624 | 0.21094 | 15.61557 |
|  |  | **2** | **0.00843** | **0.40283** | **15.09742** |
|  |  | 3 | 0.10035 | 0.34822 | 12.58304 |
| NTI | Annual Mean Temperature | 1 | -0.11906 | 0.84191 | 40.459787 |
|  |  | 2 | 0.30737 | 0.11475 | 34.326925 |
|  |  | **3** | **0.63952** | **0.02748** | **26.2549** |
|  | Annual Precipitation | 1 | -0.04539 | 0.45755 | 39.778783 |
|  |  | **2** | **0.84343** | **6.30E-04** | **19.45684** |
|  |  | 3 | 0.81753 | 0.00375 | 19.446549 |
|  | Mean Diurnal Range | **1** | **0.33992** | **0.04498** | **35.181** |
|  |  | 2 | 0.32535 | 0.10466 | 34.06389 |
|  |  | 3 | 0.23349 | 0.22853 | 33.798979 |
|  | Annual Evapotranspiration | 1 | -0.02683 | 0.40732 | 39.59968 |
|  |  | 2 | -0.12635 | 0.62928 | 39.189359 |
|  |  | 3 | -0.21256 | 0.71162 | 38.385405 |
|  | Temperature Annual Range | 1 | 0.22449 | 0.09415 | 36.792512 |
|  |  | **2** | **0.35549** | **0.08919** | **33.606934** |
|  |  | 3 | 0.26134 | 0.20688 | 33.428916 |
|  | Area | 1 | -0.10497 | 0.71326 | 40.33306 |
|  |  | **2** | **0.51671** | **0.03256** | **30.7282** |
|  |  | 3 | 0.45431 | 0.08971 | 30.401078 |
|  | Human footprint | 1 | -0.11072 | 0.75661 | 40.38492 |
|  |  | **2** | **0.6761** | **0.00802** | **26.72634** |
|  |  | 3 | 0.65909 | 0.02338 | 25.69663 |





**Figure S1** Relationships between elevation and underlying variables.


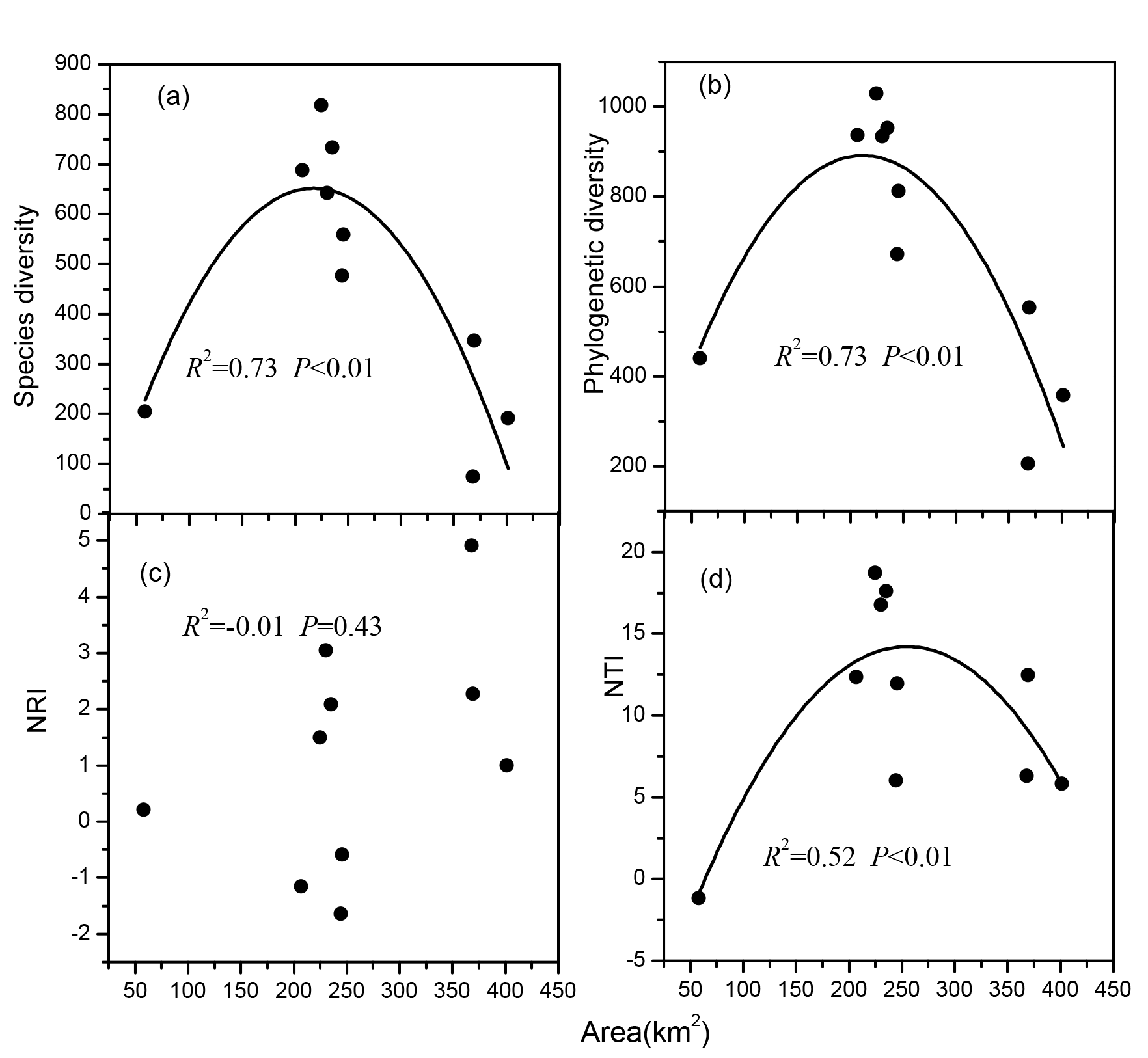


**Figure S2** Relationships between species richness (a), phylogenetic diversity (b), phylogenetic structure (c, d) and area.
